# Identification of Pathways and Key Genes in Venous Remodeling After Arteriovenous Fistula by Bioinformatics Analysis

**DOI:** 10.3389/fphys.2020.565240

**Published:** 2020-12-08

**Authors:** Kong Jie, Wang Feng, Zhao Boxiang, Gong Maofeng, Zhang Jianbin, Lu Zhaoxuan, Zhou Yangyi, Chen Liang, Su Haobo, Lou Wensheng, Chen Guoping, Gu Jianping, He Xu, Wen Jianyan

**Affiliations:** ^1^Department of Interventional Radiology, Nanjing First Hospital, Nanjing Medical University, Nanjing, China; ^2^Graduate School of Peking Union Medical College, Beijing, China; ^3^Department of Cardiovascular Surgery, China-Japan Friendship Hospital, Beijing, China

**Keywords:** hub gene, protein interaction maps, gene ontology, arteriovenous fistula, venous remodeling

## Abstract

The arteriovenous fistula (AVF) is the first choice for vascular access for hemodialysis of renal failure patients. Venous remodeling after exposure to high fistula flow is important for AVF to mature but the mechanism underlying remodeling is still unknown. The objective of this study is to identify the molecular mechanisms that contribute to venous remodeling after AVF. To screen and identify the differentially expressed genes (DEGs) that may involve venous remodeling after AVF, we used bioinformatics to download the public microarray data (GSE39488) from the Gene Expression Omnibus (GEO) and screen for DEGs. We then performed gene ontology (GO) function analysis, Kyoto Encyclopedia of Genes and Genomes (KEGG) pathway analysis, and gene set enrichment analysis (GSEA) for the functional annotation of DEGs. The protein-protein interaction (PPI) network was constructed and the hub genes were carried out. Finally, we harvested 12 normal vein samples and 12 AVF vein samples which were used to confirm the expressions of the hub genes by immunohistochemistry. A total of 45 DEGs were detected, including 32 upregulated and 13 downregulated DEGs. The biological process (BP) of the GO analysis were enriched in the extrinsic apoptotic signaling pathway, cGMP-mediated pathway signaling, and molting cycle. The KEGG pathway analysis showed that the upregulated DEGs were enriched in glycosaminoglycan biosynthesis and purine metabolism, while the downregulated DEGs were mainly enriched in pathways of glycosaminoglycan biosynthesis, antifolate resistance, and ABC transporters. The GSEA analysis result showed that the top three involved pathways were oxidative phosphorylation, TNFA signaling via NF-K B, and the inflammatory response. The PPI was constructed and the hub genes found through the method of DMNC showed that INHBA and NR4A2 might play an important role in venous remodeling after AVF. The integrated optical density (DOI) examined by immunohistochemistry staining showed that the expression of both INHBA and NR4A2 increased in AVF compared to the control group. Our research contributes to the understanding of the molecular mechanism of venous remodeling after exposure to high fistula flow, which may be useful in treating AVF failure.

## Introduction

Incidences of chronic kidney disease (CKD) and end-stage renal disease (ESRD) are rising due to the aging population and the increasing prevalence of diabetes (Vachharajani et al., 2014). Hemodialysis (HD) is the treatment of choice for ESDR patients, and over 1.5 million people worldwide receive regular HD (Grassmann et al., 2005). Well-functioning vascular access is crucial for hemodialysis (HD) patients and the ideal permanent vascular access for HD is through the native arteriovenous fistula (AVF) (Roy-Chaudhury et al., 2001; Grassmann et al., 2005; Vascular Access Work Group, 2006; Sadaghianloo et al., 2015). The AVF connects high blood pressures arterial blood flow directly into the vein, exposing the vein to disturbed flow patterns, resulting in a thickened vein wall. The outflow vein is dilated and its wall is thickened due to increased blood flow after the construction of autogenous AV access (Dixon, 2006). Although the outflow vein commonly matures for HD 3 to 4 months after creation, almost 60% of AVFs fail to mature sufficiently. Our poor understanding of the molecular mechanism of venous remodeling after the construction of AVF limits our ability to predict or prevent the non-maturation of AVF (Dixon, 2006; Dember et al., 2014).

The prevention of AVF non-maturation may benefit from compressive molecular profiling studies that reveal dysregulated genes and pathways underlying AVF non-maturation. Many researchers have tried to study the molecular mechanism involved in AVF failure. For example, Martinez reported that pro-inflammatory genes, such as CSF3R, FPR1, S100A8, S100A9, and VNN2, were upregulated in the pre-access veins just before AVF failed (Martinez et al., 2019). But this study did not report the changed genes before and after the construction of AVF (Martinez et al., 2019). Xie et al. (2019) reported that LMO7 could suppress the vascular fibrotic responses through the TGF-β pathway. The cellular events causing arterialization of the vein may serve as potential targets for the prevention of AVF non-maturation. However, the exact underlying mechanism of AVF non-maturation has not yet been completely understood. It is also crucial to identify novel molecular biomarkers for individualized therapy and early diagnosis. Chip technology has the capacity to detect all the genes within a sample to screen for differentially expressed genes (DEGs), so it could improve the understanding of AVF non-maturation.

We downloaded the array data of GSE39488 from the Gene Expression Omnibus (GEO)^1^ to study the differentially expressed genes (DEGs) between AVF samples and control samples. R was used to identify DEGS, and conduct the enrichment analyses, including gene ontology (GO) analysis, Kyoto Encyclopedia of Genes and Genomes (KEGG) pathway enrichment analysis, and gene set enrichment analysis (GSEA) (Martinez et al., 2019). Furthermore, the protein-protein interaction (PPI) of DEGs was constructed; hub genes with a high degree of DNMC were identified by the software Cytoscape with plug-in cytoHubba. Our results may aid in the design of subsequent experimental studies of AVF, and contribute to the understanding of the molecular mechanisms associated with the development of AVF failure.

## Materials and Methods

### Microarray Data

The gene expression profile of GSE39488 was downloaded from the Gene Expression Omnibus database (GEO) (see footnote). That dataset is based on the GPL10332 platform (Agilent-026652 Whole Human Genome Microarray 4 × 44K v2). The GSE39488 dataset contained 10 samples, including 4 control samples and 6 AVF samples.

### Intra-Group Data Repeatability Test

Repeatability between the two groups was evaluated by the Pearson’s correlation test. All statistical computing and graphics were performed using the R software. A heat map of correlation was drawn to visualize the correlations between all samples in the dataset using the pheatmap package of R. Principal component analysis (PCA) was performed to visualize the gene expression to assess sample relationships and variability.

### Identification of DEGs

Bioconductor project^2^ was used for the analysis and comprehension of high-throughput genomic data while the significantly differentially expressed genes (DEGs) between AVF samples and control samples were identified by the Bioconductor package limma. A *t*-test in the limma package was used to calculate the *p*-values of DEGs. The adjusted *p*-value cutoff was set at 0.05 and the | logFC| > 0.5 was the filter criteria. A set of 45 DEGs were found from 22,683 genes, including 32 upregulated and 13 downregulated genes. A heatmap of DEGs was generated in R with the R package “pheatmap.”

### Functional Annotation for DEGs Using GO, KEGG, and GSEA Analysis

The clusterProfiler package is a tool used to implement methods when analyzing and visualizing the functional profiles of genomic coordinates and was used to perform GO, KEGG, and GSEA analysis (Yu et al., 2012). Gene ontology (GO) analysis is a useful method which includes three key biological aspects: BP (biological process), MF (molecular function), and CC (cellular component). KEGG is a commonly used bioinformatics database^3^ which analyzes gene functions and enriched genes with their pathways (Altermann and Klaenhammer, 2005). The GO and KEGG enrichment analyses were performed for DEGs by the clusterProfiler package with the *p*-value cutoff and the *q*-value cutoff set to 0.01 and 0.05, respectively (Yu et al., 2012). Gene set enrichment analysis (GSEA) is a computational method to find the statistically significant difference between biological samples in a previously defined set of genes and was also performed using the clusterProfiler package.

### Construction and Analysis of the PPI Network and Identification of Hub Genes

The online Search Tool for the Retrieval of Interacting Genes (STRING) database^4^ was used to predict the PPI (protein-protein interaction) network from the imported DEGs (Szklarczyk et al., 2015). Cytoscape^5^ (Smoot et al., 2011) was used to visualize the PPI network. The plug-in cytoHubba was used to identify the hub genes through the score of DNMC. The top 10 genes with a high degree of DNMC were selected as hub genes.

### Immunocytochemistry

This study was approved by both the Medical Ethics Committee of the Nanjing First Hospital, China (KY20190823-22) and the Medical Ethics Committee of the China-Japan Friendship Hospital of Beijing, China (2019-25-1) and received informed consent from all of the patients. A 1-cm segment of the venous end distal to the anastomosis was collected as the AVF sample during the operation of the repair of the AVF. The normal veins were pieces of the great saphenous vein collected from patients who underwent coronary artery bypass grafting. The Declaration of Helsinki was strictly followed. Both the normal vein and the AVF vein samples were collected during the operation and were dehydrated and embedded in paraffin. After that, the samples were sectioned into 5 μm thick pieces, and were dewaxed, rehydrated, and stained with hematoxylin and eosin sequentially until transparent. Afterward, the sections were deparaffinated, blocked, and incubated with the primary antibody Anti-Inhibin beta A antibody (INHBA, abcam) or Anti-Nurr1 antibody (NR4A2, abcam) at 4°C overnight. The expression of both INHBA and NR4A2 were calculated as the integrated optical density (DOI) of the area which was stained yellow-brown by the Image-Pro Plus 6.0 (IPP 6.0, Media Cybernetics, United States). The DOI of both groups were present as mean and standard deviation (SD) and the independent *t*-test was performed by R software version 3.6.2 to analyze the difference between the two groups. P < 0.05 was considered as statistically significant.

## Results

### Validation of the Dataset

**FIGURE 1:**
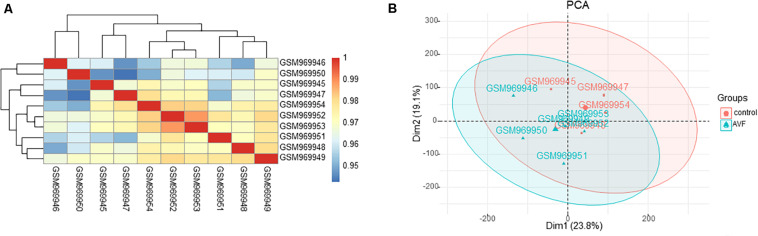
Intra-sample data repeatability test for GSE39488 by the Pearson’s correlation analysis and PCA. **(A)** All samples from the GSE39488 dataset were analyzed through Pearson’s correlation analysis. The correlation coefficient were reflected by the colors. **(B)** All samples from the GSE39488 dataset were analyzed using PCA. The PC1 and PC2 are represented on the *X*-axis and *Y*-axis, respectively. PCA, principal component analysis; PC1, principal component 1; PC2, principal component 2.

To further validate the dataset, we used a Pearson’s correlation test between different samples and applied the principal component analysis (PCA) to test the potential associations between the AVF samples and control samples. Based on the Pearson’s correlation test, the heatmap of correlation in the GSE39488 dataset showed there were strong correlations among the control samples and the AVF samples (Figure 1A). The PCA of the GSE39488 showed that the intra-group data repeatability was acceptable. The distances between the AVF samples was close to the distance between control samples in both PC1 and PC2 (Figure 1B).

### Identification of DEGs

**FIGURE 2:**
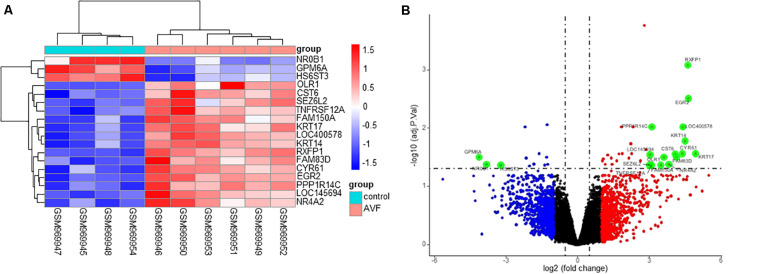
Identification of DEGs between control samples and AVF samples. **(A)** A heatmap showing the DEGs between two groups. **(B)** A Volcano plot showing the DEGs between two groups after analysis of the GSE39488 dataset with the R software. The *X*-axis represents the | logFC| and the *Y*-axis represents the *p*-value (log-scaled). FC, fold change.

A total of 45 DEGs were found based on the criteria of an adjusted *p*-value of less than 0.05 and a | logFC| value of more than 0.5, with 32 upregulated and 13 downregulated genes. Both the heatmap (Figure 2A) and Volcano plots (Figure 2B) were used to illustrate the DEGs.

### Functional and Pathway Enrichment Analysis

**FIGURE 3:**
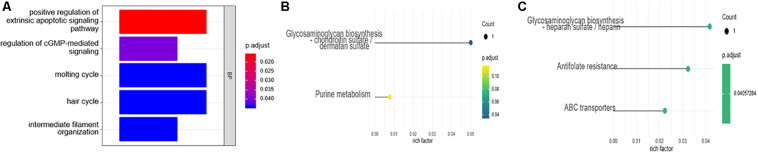
The enrichment analysis of the DEGs using the GO and KEGG analyses. **(A)** The biological process (BP) of the GO analysis. **(B)** The results of the KEGG pathways analysis of the upregulated DEGs. **(C)** The results of the KEGG pathways analysis of the downregulated DEGs.

**FIGURE 4:**
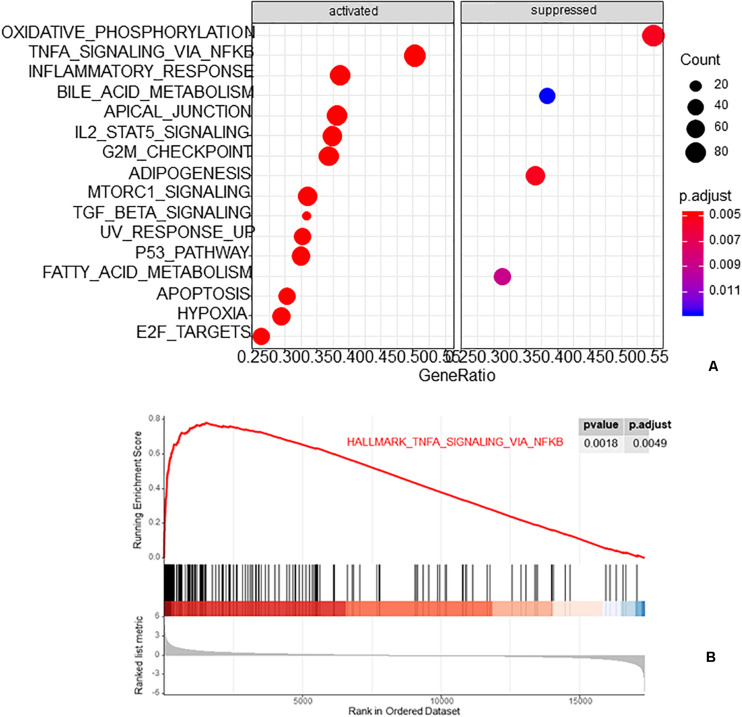
The results of gene set enrichment analysis (GSEA). **(A)** The top three pathways/gene sets enriched by GSEA. **(B)** The NF-κB pathway was enriched by GSEA.

The R package clusterProfiler was used to perform the biological functions and pathways analyses including GO, KEGG, and GSEA. Both the upregulated and the downregulated DEGs were enriched by GO categories, respectively, but no GO term was enriched by the downregulated DEGs, this may be because there were only 13 downregulated DEGs. The results of the biological process (BP) GO category showed that the upregulated DEGs were enriched in the extrinsic apoptotic signaling pathway, cGMP-mediated pathway signaling, and molting cycle (Figure 3A) while there was no GO term enriched in both the cellular component (CC) and molecular function (MF) categories. The results of the KEGG pathways analysis showed that the upregulated DEGs were mainly enriched in the pathways of glycosaminoglycan biosynthesis and purine metabolism (Figure 3B) while the downregulated DEGs were mainly enriched in the pathways of glycosaminoglycan biosynthesis, antifolate resistance, and ABC transporters (Figure 3C). The results of gene set enrichment analysis (GSEA) showed that the top three pathways/gene sets were significantly downregulated between the AVF and control samples including in oxidative phosphorylation, TNFA signaling via NF-κB, and the inflammatory response (Figure 4A). We found four DEGs belonging to the NF-κB pathway, those were NR4A2, INHBA, EGR2, and OLR1 (Figure 4B).

### Construction of the Protein-Protein Interaction (PPI) Network and the Selection of Hub Genes

**FIGURE 5:**
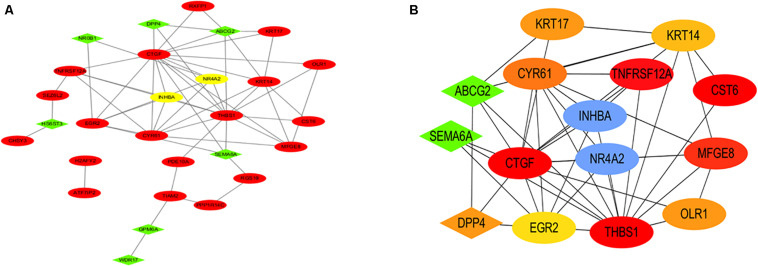
PPI network and hub genes. **(A)** The PPI network analyzed by the STRING database is presented. **(B)** The hub genes included INHBA, NR4A2, TNFRSF12A, MFGE8, CYR61, KRT17, OLR1, DPP4, KRT14, and ABCG2.

The interactive relationships among DEGs were analyzed by the STRING database (see foot note) (Search Tool for the Retrieval of Interacting Genes) to construct the PPI network (Szklarczyk et al., 2015). The result of the PPI network is presented by the Cytoscape software in Figure 5A. The plug-in cytoHubba of the Cytoscape software was used to find hub genes by the method of DMNC (Figure 5B) and the top ten hub genes were INHBA, NR4A2, TNFRSF12A, MFGE8, CYR61, KRT17, OLR1, DPP4, KRT14, and ABCG2.

### Demographic Data of the Patients and the Expression of Both INHBA and NR4A2

**FIGURE 6:**
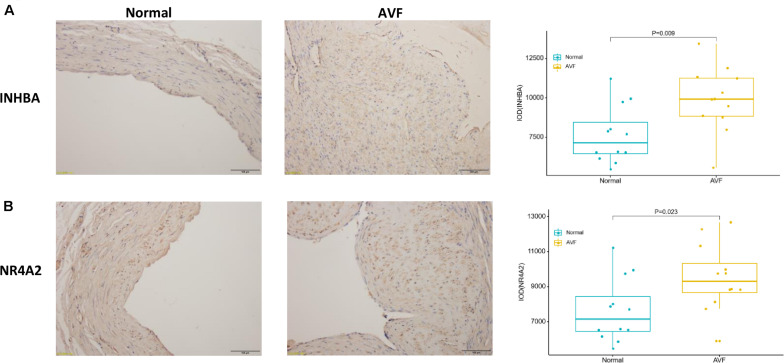
The result of immunohistochemistry staining showed that the expression of INHBA and NR4A2 were increased in AVF. **(A)** A typical representative photomicrograph of immunohistochemistry staining for INHBA. A box plot was used to show that the expression of INHBA significantly increased in AVF. **(B)** A typical representative photomicrograph of immunohistochemistry staining for NR4A2. A box plot was used to show that the expression of NR4A2 significantly increased in AVF.

Patient clinical characteristics including age, gender, hypertension, diabetes mellitus, hyperlipidemia, smoking, and BMI were collected and compared between groups. The one-stage AVF s was performed in our center so we did not require the removement of venous segments. As a result, the normal veins were collected for the control group. The results presented in Table 1 show that all of the clinical characteristics were not statistically different between the two groups. Immunohistochemistry was used to assess the expression of both INHBA and NR4A2 (Figure 6). The integrated optical density (DOI), calculated by the IPP 6.0, was used to examine the expression of the protein. INHBA was significantly higher in the AVF group (9897.19 ± 2034.88, *n* = 12) compared to the normal group (7641.65 ± 1818.00, *n* = 12), and the *p*-value was 0.009. NR4A2 was significantly higher in the AVF group (9513.74 ± 1925.76, *n* = 12) compared to the normal group (7641.65 ± 1818.00, *n* = 12), and the *p*-value was 0.023.

**TABLE 1:** Demographic data of patients.

	**Normal**	**AVF**	***P*-value**
	**(*n* = 12)**	**(*n* = 12)**	
**Age**			
Mean (SD)	59.8 (9.17)	58.4 (10.1)	0.738
**Gender**			
Female	4 (33.3%)	6 (50.0%)	0.679
**Hypertension**			
Yes	10 (83.3%)	6 (50.0%)	0.194
**Diabetes mellitus**			
Yes	10 (83.3%)	8 (66.7%)	0.637
**Hyperlipidemia**			
Yes	6 (50.0%)	8 (66.7%)	0.679
**Smoke**			
Yes	2 (16.7%)	2 (16.7%)	1
**BMI**			
Mean (SD)	22.3 (1.44)	23.5 (2.75)	0.21

**AVF, arteriovenous fistula; BMI, body mass index.**

## Discussion

It is widely accepted that AVF represents the first choice for vascular access for hemodialysis (Jindal et al., 2006; Vascular Access Work Group, 2006). However, the rate of non-maturation of AVF has increased because an increasing number of elderly and frail patients are received AVF for hemodialysis (Al-Jaishi et al., 2014). Many researchers have reported a highly complex genomic landscape after the construction of AVF (Martinez et al., 2019). The molecular mechanisms that distinguish between normal veins and veins exposed to high blood flow after the construction of AVF are poorly understood, but are of major importance for combatting AVF non-maturation.

In this study, a total of 45 DEGs (32 upregulated and 13 downregulated) were recognized by comparing normal vein samples and AVF vein samples. GO biological analysis results showed that these DEGs were mainly associated with the extrinsic apoptotic signaling pathway, cGMP-mediated pathway signaling, and the molting cycle. The KEGG pathways analysis of the upregulated DEGs included glycosaminoglycan biosynthesis and purine metabolism while the downregulated DEGs included glycosaminoglycan biosynthesis, antifolate resistance, and ABC transporters. The GSEA analysis represented the top three pathways/gene sets in which the AVF samples were significantly involved in oxidative phosphorylation, TNFA signaling via NF-κB, and the inflammatory response. Many studies have reported that vein remodeling after AVF was associated with inflammatory and NF-κB pathways, this is consistent with our study (Khavanin Zadeh et al., 2015; Brahmbhatt and Misra, 2016; Viecelli et al., 2018; Chen B. et al., 2019; Martinez et al., 2019). Brahmbhatt reported that many factors, such as uremia and hypoxia, can upregulate the inflammatory response, and eventually lead to intimal hyperplasia and secondary thrombosis of AVF (Brahmbhatt and Misra, 2016). Khavanin Zadeh reported that AVF failure has a significantly higher CRP positive rate (Khavanin Zadeh et al., 2015).

Subsequently, key genes were identified by the construction of a PPI network, and the hub genes were discovered through the DMNC method. The top ten hub genes were INHBA, NR4A2, TNFRSF12A, MFGE8, CYR61, KRT17, OLR1, DPP4, KRT14, and ABCG2. The first two genes, INHBA and NR4A2, are involved in the NF-κB signaling pathway, which is consistent with the result of GSEA. INHBA (inhibin subunit beta A) is a gene that could encode a type of the TGF-beta (transforming growth factor-beta) superfamily of proteins (Fagerberg et al., 2014). Castier, Y reported that the induction of NF-κB by shear stress contributes to matrix metalloproteinases induction and affects the vascular remodeling of vascular enlargement after AVF (Castier et al., 2009). In the study of Fukasawa, M, a balloon catheter containing an NF-κB decoy oligodeoxynucleotide could sustain for a longer period of time compared to the control group (Fukasawa et al., 2019). NR4A2 (nuclear receptor subfamily 4 group A member 2) could encode one of the steroid-thyroid hormone-retinoid receptor superfamily, which may act as a transcription factor (Fagerberg et al., 2014). TNFA signaling via NF-κB was enriched by the GSEA analysis. Among these top 10 hub genes, INHBA and NR4A2 were related to the TNFA signaling pathway. So we focused on INHBA and NR4A2. Both INHBA and NR4A2 have been reported to be involved in the NF-κB pathway in many studies. Wamsley et al. (2015) reported that NF-κB activity is needed to upregulate INHBA which could maintain mesenchymal phenotypes. The research from Chen J. et al. (2019) identified NR4A transcription factors as having an important role in the lesser effect of T cells against solid tumors. Popichak KA reported that NR4A2 could suppress the inflammatory response of NF-κB. We confirmed that the expression of both INHBA and NR4A2 were increased in the AVF samples by immunohistochemistry. Both INHBA and NR4A2 may be potential therapeutic targets for the prevention of AVF. We will try to suppress these genes *in vivo* and *in vitro* in future to confirm their role in AVF.

There are several limitations in this study. Firstly, the control samples are not from uremic patients because it is difficult to obtain normal vein samples from uremic patients according to the Declaration of Helsinki. In our center, we used one-stage AVFs so we did not require the removal of the venous segments. As a result, we did not collect venous samples from uremic patients. We will try to collect normal vein samples from uremic patients for the further study. Secondly, each sample was collected at a different time after AVF surgery. The collection time depended on the patency time of the AVF. We will enlarge the sample size so that we can collect AVF samples at the same time in a future study. Thirdly, we did not isolate the endothelial cells from the smooth muscle cells and then analyze them separately. We will try to analyze different cell types in the future study.

## Conclusion

This study identified a total of 45 DEGs. GO, KEGG, and GSEA enrichment analyses were performed and the NF-κB pathway was suggested to be involved in vein remodeling. The hub genes were identified, including INHBA and NR4A2. Both of these two genes have been reported to be associated with the NF-κB pathway in many studies. Altogether, our study indicates that vein remodeling may be regulated by INHBA and NR4A2 through the NF-κB pathway. Therefore, the detailed investigation of the molecular mechanism and clinical application of these genes are needed in the future.

## Data Availability Statement

The datasets presented in this study can be found in online repositories. The names of the repository/repositories and accession number(s) can be found in the article/supplementary material.

## Ethics Statement

This study was approved by the Medical Ethics Committee of the Nanjing First Hospital, China (KY20190823-22) and the Medical Ethics Committee of the China-Japan Friendship Hospital of Beijing, China (2019-25-1) and received written informed consent from all of the parents.

## Author Contributions

GJ, WJ, and HX designed, guided, and funded the study. KJ and ZB conducted most of the experimental work. GM helped with the software calculation. LZ and ZY assisted with drawing pictures. CL helped with the revised picture. KJ, SH, LW, and CG performed the data analysis. WF performed the immunocytochemistry. KJ and GM conducted the data interpretation. KJ and ZB performed the drafting of the manuscript. GJ and HX did the critical revision of the manuscript and final approval for publication. All authors contributed to the article and approved the submitted version.

## Conflict of Interest

The authors declare that the research was conducted in the absence of any commercial or financial relationships that could be construed as a potential conflict of interest.

## Footnotes

1 http://www.ncbi.nlm.nih.gov/geo/

2 http://www.bioconductor.org/

3 https://www.kegg.jp/

4 https://string-db.org/

5 http://cytoscape.org/
